# Triplet‐Triplet Annihilation‐Based Upconversion in Rod‐Like Silica Capsules

**DOI:** 10.1002/advs.78708

**Published:** 2026-11-09

**Authors:** Jeong‐Min Park, Chanwook Cha, Hyun‐Seok Choe, Geun Young Kim, Suyoung Jeong, Han‐Gil Kim, Koohee Han, Jae‐Hyuk Kim

**Affiliations:** ^1^ Department of Civil and Environmental Engineering Pusan National University Busan Republic of Korea; ^2^ School of Chemical Engineering and Applied Chemistry Kyungpook National University Daegu Republic of Korea; ^3^ Center For Advanced Specialty Chemicals Korea Research Institute of Chemical Technology Ulsan Republic of Korea; ^4^ Institute For Environment and Energy Pusan National University Busan Republic of Korea

**Keywords:** anisotropic microparticle, anisotropic polarizability, electric‐field alignment, rod‐like silica capsule, triplet‐triplet annihilation upconversion, upconversion

## Abstract

Most reported triplet‐triplet annihilation upconversion (TTA‐UC) platforms rely on isotropic spherical particles, leaving shape‐derived functions of anisotropic carriers largely unexplored. Here, we developed rod‐like silica capsules (UC RCs) by loading hollow mesoporous silica rods with a PdTPBP/perylene/squalene‐based TTA‐UC oil and sealing the mesopores with an additional silica layer, which effectively suppressed oil leakage during ethanol exposure and dry‐powder storage. In aqueous suspension, UC RCs exhibited red‐to‐blue TTA‐UC under low‐intensity excitation, with a threshold intensity of 55 mW cm^−^^2^ and an upconversion quantum yield of 1.47%. To add spatial controllability to these efficient upconverting microcarriers, we harnessed their rod‐like geometry and investigated their electric‐field response. Coupled‐dipole calculations and rotational Monte Carlo simulations showed that shape‐derived polarizability anisotropy favors field‐parallel orientation, with an equilibrium mean angle of approximately 2.3° under the modeled electric field. Consistent with this prediction, UC RCs were aligned in a hydrogel matrix under an AC electric field while retaining their upconversion capability. To the best of our knowledge, this is the first demonstration of field‐aligned anisotropic TTA‐UC silica capsules embedded in a hydrogel matrix. These anisotropic microcarriers hold great promise for developing patterned luminescent materials, anti‐counterfeiting platforms, and advanced photonic architectures.

## Graphical abstract

Anisotropic rod‐like silica capsules are engineered to securely contain triplet‐triplet annihilation upconversion (TTA‐UC) media. Capping the mesoporous shell with a continuous silica layer provides structural stability, preventing internal oil leakage. This physical integrity maintains stable red‐to‐blue upconversion emission and facilitates the electric‐field‐directed spatial alignment of the microcarriers within hydrogels for patterned optical applications.

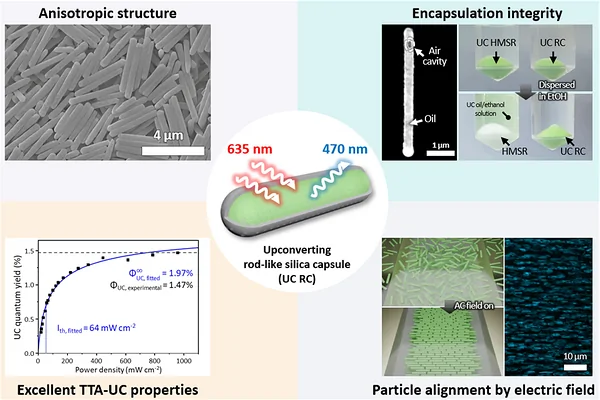

## 1 Introduction

Photon upconversion, converting lower‐energy photons into higher‐energy photons, has shown enormous potential in photonic applications, including photovoltaics, photocatalysis, and advanced biomedical imaging and therapeutics [1, 2, 3, 4]. In contrast to other upconversion mechanisms, such as second‐harmonic generation, two‐photon absorption, and lanthanide‐based upconversion, triplet‐triplet annihilation‐based upconversion (TTA‐UC) can be achieved at lower excitation intensities with a higher UC quantum yield.

TTA‐UC is implemented by intermolecular triplet energy transfer between UC chromophores (i.e., sensitizers and acceptors) [5, 6, 7]. These processes are based on molecular diffusion (i.e., Dexter energy transfer), which relies on the efficient overlap of molecular orbitals within 10 Å. During triplet energy transfer, the presence of molecular oxygen should be prevented because the triplet excited states of UC chromophores are quenched by molecular oxygen. TTA‐UC is achieved through several sequential steps: (1) formation of photosensitizers’ singlet excited states (^1^S^*^) by absorbing light, (2) conversion of ^1^S^*^ into triplet excited states (^3^S^*^) by intersystem crossing (ISC), (3) triplet energy transfer to acceptors, generating their triplet excited states (^3^A^*^), (4) generation of acceptors’ singlet excited states (^1^A^*^) by TTA between two or more excited acceptors, and (5) emission of upconverted delayed fluorescence.

For these reasons, the earlier TTA‐UC systems were implemented using deoxygenated organic solvents (e.g., toluene, tetrahydrofuran) to provide sufficient molecular diffusion, solubility of UC chromophores, and prevention of quenching by molecular oxygen [8, 9, 10]. However, their toxicity and volatility pose challenges for expanding TTA‐UC applications. Subsequently, researchers reported that unsaturated media, such as oleic acid, limonene, and squalene, enable TTA‐UC even in the presence of molecular oxygen because their reductive characteristics act as scavengers for generated singlet oxygen, creating a locally deoxygenated environment around the UC chromophores [11, 12, 13].

Recent research has focused on fabricating air‐tolerant and photostable TTA‐UC nano‐/microparticles using unsaturated media to harness the superiority of these media for broader applications [14, 15, 16]. TTA‐UC nano‐/microparticles offer various advantages, including physical stability and high‐local but low global concentration of UC chromophores, which maximize UC quantum yield and light penetration depths [17]. Despite these advances, most reported TTA‐UC microcarriers remain isotropic, predominantly spherical, which limits opportunities for spatial programming, directional assembly, and anisotropic photonic architectures. Implementing TTA‐UC in anisotropic particles could introduce shape‐derived functions such as orientation control, asymmetric assembly, and enhanced interfacial accessibility [18, 19, 20, 21].

In this study, we synthesized hollow mesoporous silica rods (HMSRs) using silica rods as sacrificial templates and employed them as anisotropic microcarriers for TTA‐UC oil encapsulation. PdTPBP/perylene/squalene‐based TTA‐UC oil was infused into the hollow interior of HMSRs to produce UC oil‐laden HMSRs (UC HMSRs). The open mesopores were subsequently sealed with an additional silica layer to yield UC oil‐laden rod‐like silica capsules (UC RCs), thereby improving the retention of the internal UC oil during handling, solvent exposure, and dry storage. The resulting UC RCs exhibited red‐to‐blue TTA‐UC emission in aqueous suspension under low‐intensity excitation. Beyond serving as stable TTA‐UC microcarriers, their anisotropic geometry provides a means to directionally organize the luminescent capsules under an external field. We therefore analyzed their field‐induced orientational tendency using the coupled‐dipole method, rotational Monte Carlo simulations, and Boltzmann‐distribution analysis. The predicted field‐parallel orientational tendency was subsequently implemented by aligning UC RCs in a hydrogel matrix under an AC electric field while retaining their upconversion functionality. This work demonstrates that anisotropic silica capsules can integrate stable TTA‐UC oil encapsulation with spatial organization, providing a route toward shape‐programmable optical microcarriers.

## 2 Results and Discussion

HMSRs were synthesized through three sequential steps: (1) preparation of a silica rod (SR) template, (2) coating of the SR with a mesoporous organosilica layer (SR@mSiO_2_), and (3) selective etching of the inner SR template (Figure 1A and Figure S1). SR was synthesized with a slight modification of a previously reported method [22]. A water emulsion was first formed in a pentanolic solution of polyvinylpyrrolidone (PVP) and stabilized using trisodium citrate dihydrate (TSC) and PVP. Subsequently, the SR grew with the addition of tetraethyl orthosilicate (TEOS) as a silica precursor. The growing silica was positioned on one side of the water emulsion and grew preferentially in one direction as the silica precursor was consumed. This anisotropic growth is generally attributed to the combined roles of TSC and ammonia in modulating the droplet–silica interfacial interaction, which promotes partial dewetting of the emulsion droplet and confines silica condensation to one side of the growing particle. A mesoporous organosilica layer was then coated onto the SR template using cetyltrimethylammonium bromide (CTAB) and 1,2‐bis(triethoxysilyl)ethane (BTSE) as the pore‐directing agent and organosilica precursor, respectively [23, 24]. Subsequently, CTAB was extracted by ion exchange using an ethanolic NH_4_NO_3_ solution, and the SR template was selectively etched using a hot NaOH solution to synthesize the HMSRs. The selective removal of the inner SR template was enabled by the higher alkaline resistance of the BTSE‐derived organosilica framework, which consists of a Si–O–Si network containing Si–CH_2_–CH_2_–Si bridges, compared with the inorganic silica framework consisting solely of a Si–O–Si network [25, 26]. Compared with the existing synthetic method employing a metal/metal‐oxide rod template for hollow mesoporous silica rods, our approach using a silica rod template has several advantages, such as cost‐effectiveness and ease of adjustment of its length up to several micrometers by varying the amount of TEOS. In addition, the use of an all‐silica template can minimize concerns associated with residual metal species after template removal.

**FIGURE 1:**
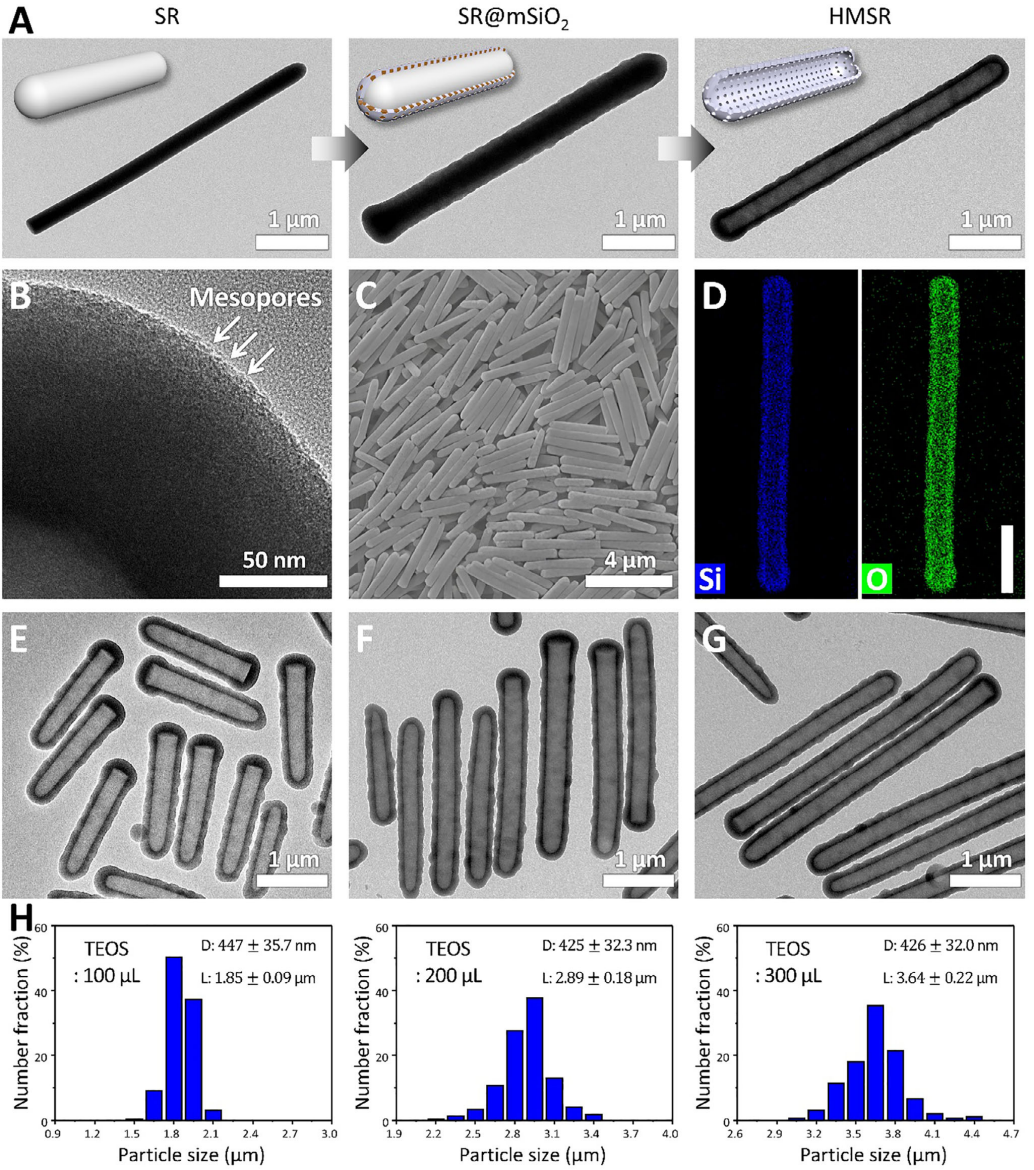
(A) TEM images for each step of HMSR synthesis. (B) High‐magnification TEM image of HMSR shell. (C) SEM image of HMSRs. (D) EDS elemental maps of HMSRs (scale bar = 1 µm). (E–G) TEM images of HMSRs with different aspect ratios obtained by varying the amount of TEOS. (H) Particle length distribution of HMSRs with different aspect ratios (n = 300).

The morphology, composition, and textural properties of the synthesized HMSRs were characterized using transmission electron microscopy (TEM), scanning electron microscopy (SEM), particle size analysis, energy‐dispersive X‐ray spectroscopy (EDS) elemental maps, X‐ray diffraction (XRD) analysis, and nitrogen sorption analysis. The HMSRs exhibited a shell thickness of approximately 100 nm, a diameter of 426 ± 32 nm, and a length of 3.64 ± 0.22 µm (Figure 1B,C). EDS elemental mapping showed uniform distributions of Si and O throughout the rod‐like shell, confirming the formation of a continuous silica‐based framework (Figure 1D). The XRD pattern of HMSRs showed a broad peak between 20° and 30°, corresponding to an amorphous silica structure (Figure S2). The nitrogen sorption isotherm of HMSRs exhibited a type IV curve, indicating its mesoporous properties, and the pore size distribution curve revealed that the average diameter of the mesopores was 2.5 nm (Figure S3) [27]. The Brunauer–Emmett–Teller (BET) surface area and pore volume of HMSRs were 1,892 m^2^ g^−1^ and 0.9 cm^3^ g^−1^, respectively. The high surface area and pore volume are consistent with the hollow interior and mesoporous organosilica shell, which are beneficial for subsequent oil loading. The aspect ratio of the HMSRs can be easily adjusted by varying the amount of TEOS (Figure 1E–G). The lengths of HMSRs increased (1.85 ± 0.09, 2.89 ± 0.18, and 3.64 ± 0.22 µm) depending on the amount of TEOS (100, 200, and 300 µL), while their diameters were almost identical (Figure 1H and Figure S4). The nearly unchanged diameter indicates that TEOS primarily contributed to axial elongation rather than radial growth. These results demonstrate that our strategy is straightforward for synthesizing HMSRs with readily tunable aspect ratios.

The high surface area, mesoporous shell, hollow interior, and tunable anisotropic geometry of HMSRs make them suitable microcarriers for TTA‐UC systems. In detail, the use of HMSRs as TTA‐UC microcarriers provides an opportunity to integrate upconversion behavior with shape‐derived functions, such as orientational control and field‐induced assembly, which are difficult to realize with conventional spherical particles.

Therefore, we utilized HMSRs to develop rod‐like TTA‐UC capsules that combine oil encapsulation, physical stability, and anisotropy‐derived functionality. To achieve this, we encapsulated red‐to‐blue upconverting oil, consisting of palladium(II) meso‐tetraphenyltetrabenzoporphyrin (PdTPBP, sensitizer), perylene (acceptor), and squalene (medium), inside the HMSR structure (Figure 2A and Figure S5). Squalene, a biocompatible polyunsaturated hydrocarbon, was selected as the medium because it can mitigate oxygen‐induced triplet quenching by scavenging reactive oxygen species generated during TTA‐UC.

**FIGURE 2:**
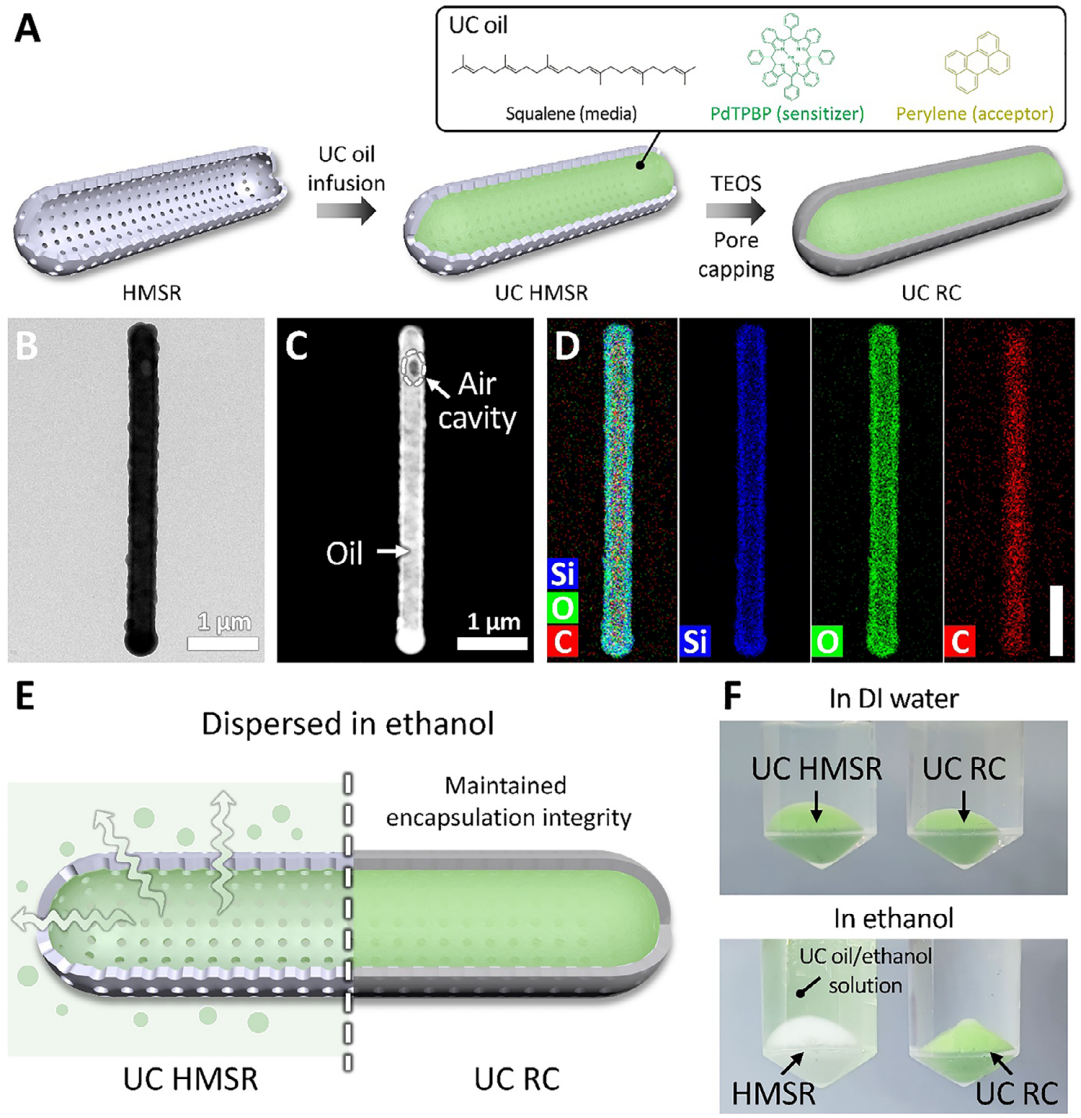
(A) Schematic illustration of the synthetic process of UC oil‐laden rod‐like silica capsules (UC RCs). (B) TEM image, (C) HAADF‐STEM image, and (D) EDS elemental maps of UC RCs (scale bar = 1 µm). (E) Schematic illustration of the function of the additional silica layer in UC RCs for suppressing UC oil leakage. (F) Photographs demonstrating the enhanced physical stability of UC RCs compared to that of UC HMSRs (control) after dispersion and centrifugation in DI water and ethanol.

Upconverting oil‐laden HMSRs (UC HMSRs) were prepared using a previously reported post‐encapsulation strategy [28]. This strategy is not specific to the PdTPBP/perylene pair and is broadly applicable to TTA‐UC systems based on molecular sensitizer–annihilator pairs. However, hybrid TTA‐UC systems employing nanoscale inorganic sensitizers, such as semiconductor quantum dots, may require alternative loading approaches because their larger dimensions could hinder transport through the mesoporous shell [29, 30]. The HMSRs were dispersed in the upconverting oil and mixed to infuse the oil into the inner space. The oil/HMSR mixture was then transferred into an aqueous ammonia solution under vigorous mixing, which promoted migration of the oil‐infused HMSRs from the oil phase to the aqueous phase owing to the hydrophilic outer silica surface, while ammonia assisted in destabilizing the Pickering emulsion. Repeated centrifugation and resuspension removed residual external UC oil, yielding an aqueous dispersion of UC HMSRs. The successful infusion of the UC oil was preliminarily verified by the observation of TTA‐UC emission from the as‐prepared UC HMSRs, which is consistent with our previous studies using hollow mesoporous silica nano/microparticles [15, 28]. This observation indicates that the approximately 100‐nm‐thick mesoporous organosilica shell does not prevent 635 nm excitation light from reaching the encapsulated sensitizer or the resulting upconverted emission from escaping the particles.

Nevertheless, UC HMSRs suffered from limited physical stability during morphological analysis and dry storage because the open mesopores allowed direct contact between the encapsulated oil and the external environment. To solve these problems, blocking the open mesopores of the UC HMSRs was necessary to isolate the inner UC oil from the external environment. Therefore, we synthesized mesopore‐capped UC HMSRs by introducing an additional silica layer on the surface of the UC HMSRs (UC oil‐laden rod‐like silica capsules, UC RCs) (Figure 2A and Figure S6). It should be noted that this capping process was conducted in the absence of ethanol to prevent any dissolution of the inner UC oil into the outer solution. Silica nanoparticles are typically synthesized using the Stöber method, which requires alcohol‐rich conditions. However, even in the absence of an alcoholic solution, the mesopores were successfully capped without particle aggregation or oil leakage. This successful capping was confirmed by TEM, high‐angle annular dark‐field STEM (HAADF‐STEM), and EDS analyses (Figure 2B–D). Compared with the HMSR, UC RCs exhibited a solid‐rod‐like contrast in TEM image (Figure 2B); however, the HAADF‐STEM image and EDS mapping showed an enhanced carbon signal localized in the inner region, supporting the encapsulation of squalene‐based UC oil (Figure 2C,D). In the case of UC HMSRs, these images could not be obtained because of UC oil leakage under the vacuum conditions of the TEM (data not shown).

The practical advantage of this capping process was further demonstrated by comparing the stability of UC HMSRs and UC RCs in deionized (DI) water and ethanol (Figure 2E,F). The encapsulated oil in UC HMSRs leached out in ethanol, resulting in a white precipitate and a light‐green supernatant (i.e., HMSRs and dissolved UC oil in ethanol). In contrast, the encapsulated oil in the UC RCs was retained even in ethanol because the additional silica layer effectively isolated the inner UC oil from the external solvent. Moreover, the capped mesopores enabled storage of UC RCs as a dried powder for at least 6 months without apparent oil leakage (Figure S7). Thus, the capping step converted the open mesoporous carrier into a sealed rod‐like capsule, while preserving the rod morphology. This capability resolves a critical preservation issue that is generally challenging for oil‐laden porous materials. This physical integrity is a critical prerequisite for reliable optical characterization, ensuring that the TTA‐UC performance is not diminished by component leakage or environmental exposure. Therefore, UC RCs were used for subsequent optical characterization because their sealed structure minimizes component leakage during dispersion, handling, and measurement.

The TTA‐UC behavior of the aqueous UC RC suspension was first examined under white light, UV irradiation, and 625 nm LED excitation by comparison with control capsules, including squalene‐laden RCs, PdTPBP RCs, and perylene RCs (Figure 3A). The perylene RC and UC RC suspensions emitted intense blue fluorescence corresponding to the Stokes emission of perylene under UV irradiation (Figure 3A, center). In contrast, only the UC RC suspension showed anti‐Stokes blue emission even under low‐power 625 nm LED excitation (23.3 mW cm^−^^2^) owing to the co‐presence of PdTPBP and perylene (Figure 3A, right). This emission was readily visible to the naked eye through a 500 nm short‐pass filter. This anti‐Stokes emission arises from triplet energy transfer from photoexcited PdTPBP to perylene acceptors, followed by triplet‐triplet annihilation between excited perylene molecules and delayed fluorescence from the singlet‐excited perylene state (Figure S5). The UC characteristics were also identified using a customized fluorescence microscope equipped with a 635 nm laser excitation source and an appropriate optical filter to visualize the upconverted emission (Figure 3B). The microscopy images further showed that blue upconverted emission was localized at individual UC RCs, rather than in the surrounding aqueous phase. These results confirm that TTA‐UC was successfully achieved in UC RCs and are consistent with the retention of the UC medium inside the sealed capsules.

**FIGURE 3:**
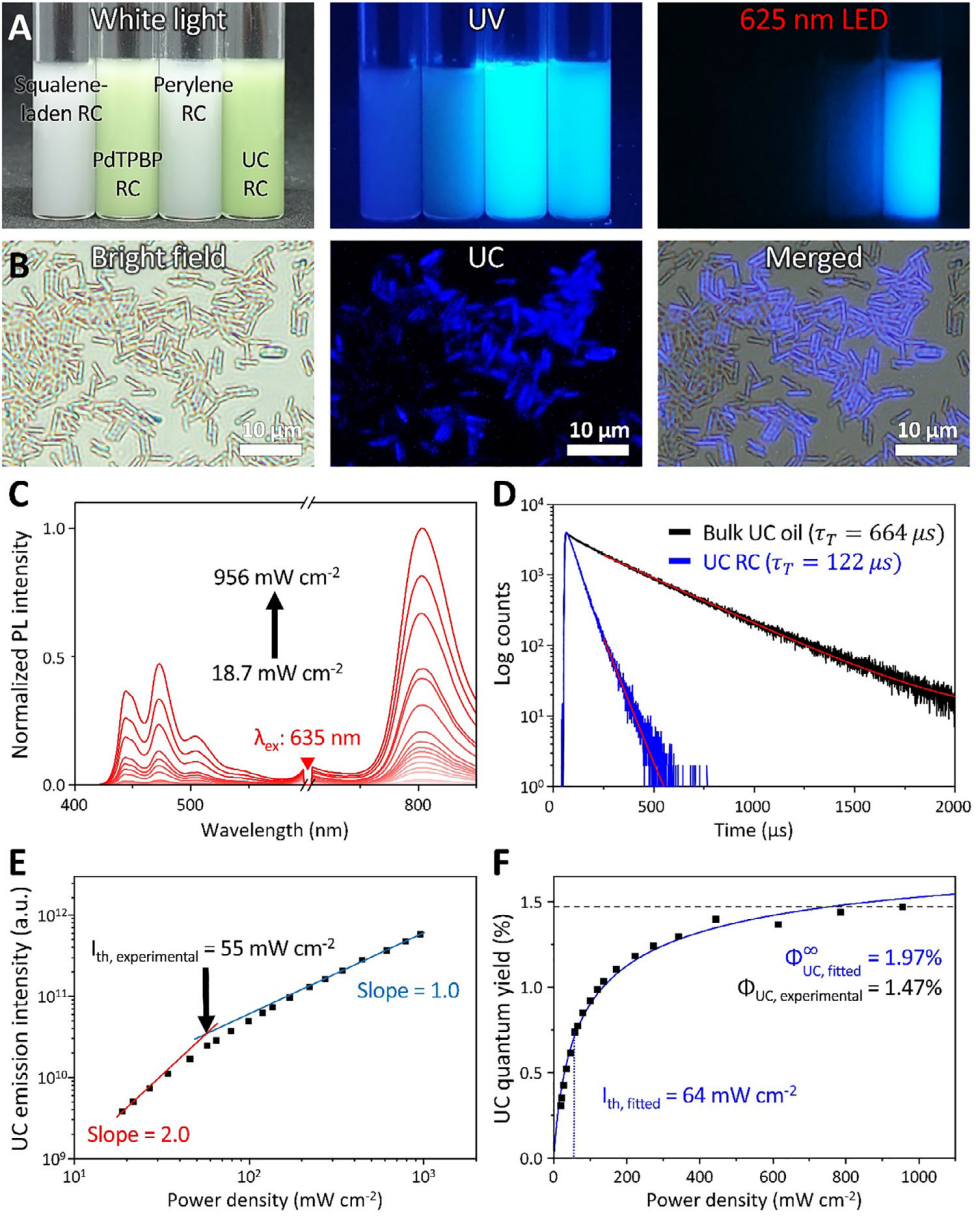
(A) Photographs of squalene‐laden RCs, PdTPBP RCs, perylene RCs, and UC RCs under white light, ultraviolet (UV), and 625 nm light‐emitting diode (LED) excitation. The images under UV and 625 nm LED excitation were acquired through a 500 nm short‐pass filter. (B) Bright‐field, UC, and merged microscopy images of UC RCs under 635 nm laser excitation (barrier filter wavelength: 432–482 nm). (C) Emission spectra of UC RCs with increasing 635 nm laser excitation. (D) UC emission decay profiles of the bulk UC oil and aqueous UC RC suspension at 470 under 635 nm laser excitation. (E) Integrated UC emission intensity and (F) UC quantum yields of UC RCs as a function of the power density of the 635 nm laser excitation. A 632 nm notch filter was used to remove the scattered incident light.

For the quantitative analysis of TTA‐UC properties of UC RCs, we measured their photoluminescence spectra, UC lifetime, UC emission intensity, and UC quantum yield (Figure 3C–F). Unlike the typical emission profile of a dilute perylene solution, where the peak at approximately 440 nm is dominant, the UC emission of UC RCs exhibited an altered spectral profile with the maximum intensity appearing at 470 under 635 nm laser excitation (Figure 3C). This change in relative peak intensity is likely associated with reabsorption/self‐absorption and inner‐filter effects arising from the high concentration of perylene in UC oil. Such a high acceptor concentration is required to promote efficient triplet energy transfer from PdTPBP to perylene and subsequent acceptor‐acceptor triplet diffusion. Despite this high concentration, perylene remains in a monomeric state in the squalene medium without forming physical aggregates or excimers. As shown in the UV‐Vis absorption and fluorescence spectra of the individual chromophore solutions (Figure S8), the system retains the characteristic monomeric peaks of perylene and exhibits no broad excimer emission band at approximately 550 nm, confirming that the spectral alteration originates solely from the optical self‐absorption effect.

The perylene triplet lifetime (τ_T_) of the UC RCs was obtained to be 122 µs by the tail fitting of the UC emission decay profile according to the relationship, I_UC_(t) ∝ exp(−t/τ_UC_) = exp(−2t/τ_T_), where τ_UC_ is the UC emission lifetime (Figure 3D) [31]. The shorter τ_T_ of UC RCs compared with that of bulk UC oil (664 µs) suggests enhanced nonradiative triplet deactivation after encapsulation, likely due in part to oxygen quenching through the silica shell and the larger interfacial area exposed to the aqueous phase. This shortened triplet lifetime was further corroborated by excitation‐power‐dependent UC emission decay measurements over 22.55–187.25 mW cm^−^^2^ (Figure S9). A global fit of the decay profiles to the TTA decay model yielded a common τ_*T*_ of 128.82 µs, consistent with the value obtained from the tail analysis. The fitted β value increased from 0.00 to 0.14 with increasing excitation intensity, distinguishing the UC RCs from nano‐confined TTA systems showing nearly excitation‐intensity‐independent decay kinetics [32]. The relatively small β values may also reflect enhanced first‐order triplet deactivation, while the coexistence of the large hollow interior and mesoporous regions may contribute to an intermediate kinetic behavior.

The UC emission intensity of UC RCs increased with increasing incident laser power density (Figure 3E). In the double logarithmic plot of the UC emission intensity against the laser power density, the UC emission intensity gradually transitioned from a quadratic to a linear dependence with increasing laser power density. As reported in previous studies, this transition indicates that the TTA process becomes increasingly dominant compared to pseudo‐first‐order decay for the depopulation of acceptor excited triplet states as the laser power density increases. The crossover point between the quadratic and linear regimes (i.e., threshold intensity, *I*_th, experimental_) of the UC RCs was determined to be 55 mW cm^−^^2^, indicating that effective TTA‐UC occurred at relatively low excitation intensity. Similarly, the UC quantum yield of the UC RCs increased with increasing laser power density (Figure 3F). The theoretical maximum UC quantum yield (Φ_UC_) is 50% because the upconverted photon is generated from two lower‐energy photons. The highest UC quantum yield of the UC RCs was measured to be 1.47% (detailed in Text S1), which is comparable to a high reported value for TTA‐UC nanoparticles using the identical PdTPBP/perylene pair (1.6%) [33]. Notably, this high efficiency was achieved despite the shorter triplet lifetime of UC RCs compared with that of the bulk oil, which suggests partial triplet deactivation after encapsulation. In general, anisotropic structures possess a higher surface‐to‐volume (S/V) ratio than isotropic spheres of the identical volume. However, owing to the micrometer‐scale dimensions of the UC RCs, their S/V ratio was approximately 32.5% of that of reported TTA‐UC particles with a diameter of ∼200 nm, which provides a structural advantage in suppressing oxygen influx (detailed in Text S2). Despite their micrometer‐scale lengths, UC RCs remained well dispersed in aqueous media during optical measurements, enabling reproducible TTA‐UC characterization.

Based on the UC quantum yield, the maximum UC quantum yield of this system and I_th_ were obtained by curve fitting with the theoretical equation: [34]

$$\;\Phi_{\mathrm{UC}}=\Phi_{\mathrm{UC}}^{\infty }\;\left\{1+\frac{1-\sqrt{1+4\left(I_{\mathrm{ex}}/I_{\mathrm{th}}\right)}}{2\left(I_{\mathrm{ex}}/I_{\mathrm{th}}\right)}\right\}$$

where Φ_UC_, $\Phi_{UC}^{\infty }$, *I*_th_, and *I*_ex_ denote the UC quantum yield, maximum UC quantum yield for the strong excitation limit of the system, threshold intensity, and laser power density, respectively. Curve fitting yielded $\Phi_{\mathrm{UC}}^{\infty }$ and *I*_th_ values of 1.97% and 64 mW cm^−2^, respectively. The fitted *I*_th_ value is in reasonable agreement with the experimentally estimated crossover intensity of 55 mW cm^−^^2^. These results indicate that the experimental data closely correspond to the theoretical equation.

Overall, UC RCs exhibited efficient TTA‐UC performance in aqueous suspension, as evidenced by anti‐Stokes emission under red excitation, characteristic UC decay kinetics, and power‐density‐dependent UC emissions. Importantly, the anisotropic rod‐shaped geometry can provide shape‐derived advantages that are difficult to achieve with isotropic spherical TTA‐UC nanoparticles, including the potential for orientation control under flow or external fields and, at an equivalent volume, a larger accessible surface area for surface functionalization. Together with their anisotropic morphology and physical stability in aqueous media, UC RCs may serve as versatile microcarriers for integration into diverse photonic and functional material applications. This combination of excellent optical performance and anisotropic geometry motivated the subsequent exploration of field‐induced spatial organization.

In principle, rod‐like anisotropic particles exhibit preferential orientation under an electric field because their polarizability differs along the longitudinal and transverse axes, resulting in an orientation‐dependent induced dipole moment. To rationalize the electric‐field‐induced alignment of UC RCs, we used the coupled‐dipole method (CDM) to estimate the anisotropic excess polarizability of an effective rod‐like particle and to derive its orientation‐dependent excess potential energy (detailed in Text S3) [35]. A UC RC was modeled as an effective dielectric rod with spatially homogeneous material properties, while the difference between its longitudinal and transverse polarizabilities arose from the anisotropic rod‐like geometry.

In the CDM, the effective rod was represented by mutually interacting polarizable units (Figure 4A). Their induced dipole moments were calculated self‐consistently by accounting for both the applied electric field and the fields generated by the other units. The resulting collective excess polarizability tensor, α_*c*_, was diagonalized to obtain the longitudinal and transverse principal polarizabilities, α_*c*,∥_ and α_*c*,⊥_, respectively. The orientation‐dependent excess potential energy, *V_E_* (θ), was expressed as

$$V_{E}\;\left(\theta \right)=-\frac{1}{2}\left(\alpha_{c,∥}-\alpha_{c,⊥}\right)E^{2}\cos^{2}\theta +\mathrm{const}$$

where *E* is the electric‐field magnitude used in the calculation and θ is the angle between the rod axis and the electric‐field direction. Because the modeled rod exhibited α_*c*,∥_ > α_*c*,⊥_, the field‐parallel orientation corresponds to the minimum‐energy configuration.

**FIGURE 4:**
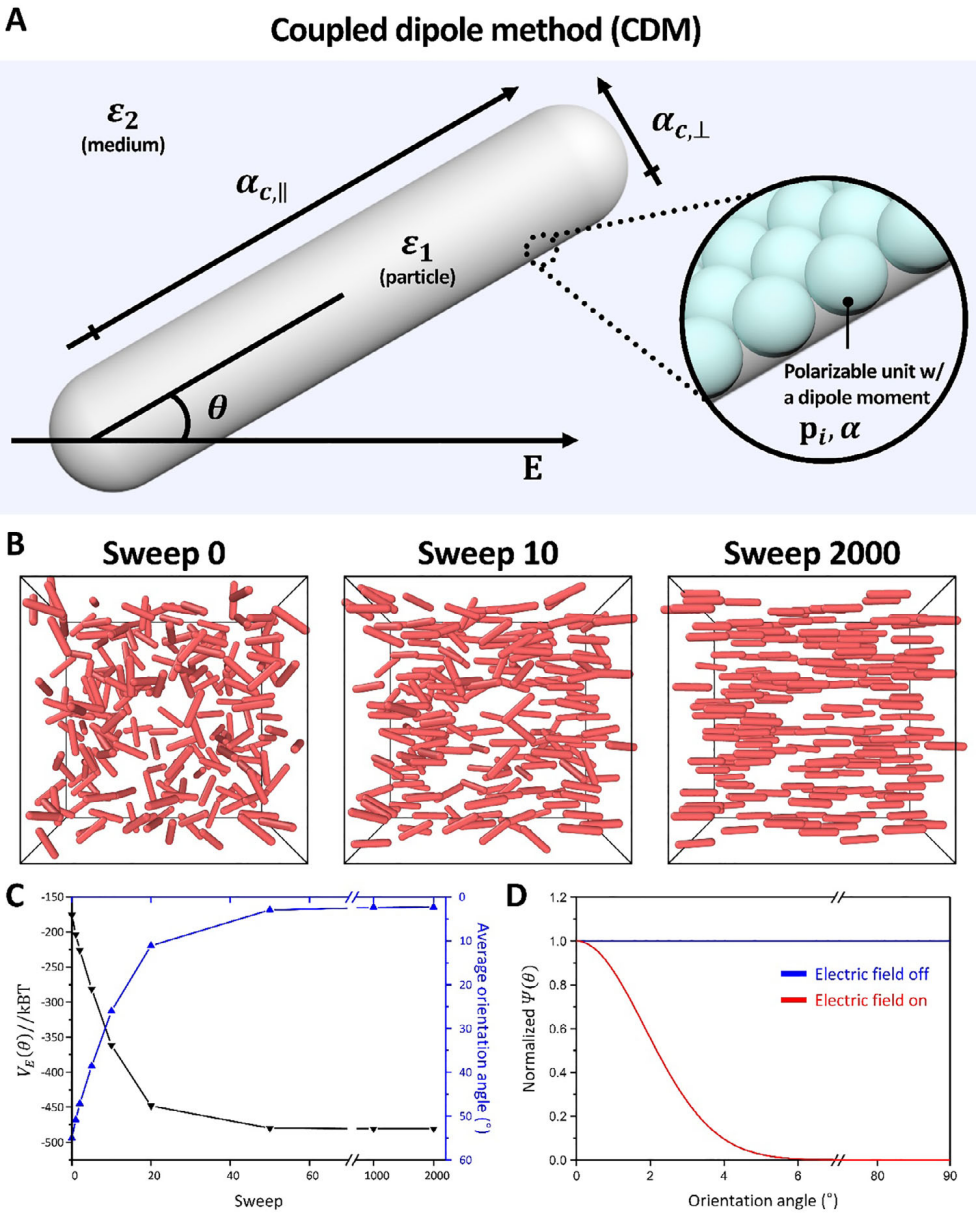
(A) Schematic illustration of the coupled‐dipole method (CDM) used to calculate the longitudinal and transverse collective polarizabilities of an effective dielectric rod. (B) Representative configurations obtained from rotational Monte Carlo simulations at 0, 10, and 2000 sweeps, showing the transition from initially random orientations toward field‐parallel alignment. (C) Average dimensionless excess potential energy and average orientation angle as a function of the number of Monte Carlo sweeps. (D) Normalized orientational distribution functions under electric‐field‐off and electric‐field‐on conditions, calculated from the CDM‐derived potential energy.

The CDM‐derived potential energy was subsequently used in rotational Monte Carlo simulations to examine the evolution of initially disordered UC RC orientations under the applied field (Figure 4B). Trial rotational moves were accepted or rejected according to the Metropolis criterion,

$$\;P_{acc}=\min\left[1,\exp\left(-\beta \Delta V_{E}\right)\right]$$

where

$$\Delta \;V_{E}=V_{E}\;\left(\theta_{new}\right)-V_{E}\left(\theta_{old}\right),\;\;\;\;\;\beta =\frac{1}{k_{B}T}$$

Here, *P*_acc_ is the probability of accepting the proposed rotational move, Δ*V_E_* is the change in potential energy between the new and old orientations, θ_new_ and θ_old_ are the proposed and previous orientation angles, respectively, β is the inverse thermal energy, *k_B_* is the Boltzmann constant, and *T* is the absolute temperature. Trial rotations that decreased the excess potential energy were therefore energetically favored, whereas rotations that increased the energy could still be accepted with a finite thermal probability. As the number of Monte Carlo sweeps increased, the average excess potential energy decreased sharply during the initial stage and subsequently approached a steady value (Figure 4C). Concurrently, the average orientation angle decreased from the initially isotropic value of approximately 57° to approximately 2.3° after 2000 sweeps. These simultaneous changes indicate that the initially disordered rods evolved toward energetically favored field‐parallel orientations. To determine whether the equilibrium state sampled by the Monte Carlo simulation was consistent with the thermodynamic prediction, the orientational distribution function, Ψ(θ), was calculated from the Boltzmann weight of $V_{E}\left(\theta \right)$ (Figure 4D):

$$\Psi \;\left(\theta \right)=\frac{\exp\left[-\beta V_{E}\left(\theta \right)\right]}{\int_{0}^{\pi /2}d\theta \;\sin\theta \;\exp\left[-\beta V_{E}\left(\theta \right)\right]}$$

To facilitate a clearer comparison of their angular dependence, each calculated Ψ(θ) curve was independently divided by its maximum value. Under the field‐off condition, the peak‐normalized Ψ(θ) remained constant at 1 over the entire angular range, indicating that no orientation was preferentially favored. In contrast, under the field‐on condition, the peak‐normalized Ψ(θ) exhibited its maximum at θ  = 0°  and decreased rapidly with increasing θ, indicating an increasing preference for orientations closer to the electric‐field direction. It should be noted that Ψ(θ) describes the orientation‐dependent statistical preference per unit three‐dimensional angular measure and does not directly represent the probability density with respect to the polar angle θ. Because the number of possible orientations within an interval *d*θ is proportional to sinθdθ, the corresponding polar‐angle probability density (*p*_θ_(θ)) is given by

$$\begin{array}{c} p_{\theta }\;\left(\theta \right)=\sin\theta \;\;\Psi \left(\theta \right) \end{array}$$

Accordingly, the three‐dimensional mean orientation angle under the applied electric field was calculated as:

$$\begin{array}{c} \;\langle \theta \rangle =\frac{\int_{0}^{\pi /2}\theta \sin\theta \;\Psi \left(\theta \right)\;d\theta }{\int_{0}^{\pi /2}\sin\theta \;\Psi \left(\theta \right)\;d\theta }\; \end{array}$$

This calculation yielded a mean orientation angle of 2.32°. The resulting value of 2.32° closely agreed with the equilibrium mean angle of approximately 2.3° obtained from the rotational Monte Carlo simulation. This agreement demonstrates the consistency between the equilibrium orientational state sampled by the Monte Carlo simulation and that predicted by the CDM‐based Boltzmann analysis. Taken together, the CDM calculation, Monte Carlo relaxation behavior, and Boltzmann‐distribution analysis support that the electric‐field‐induced alignment of UC RCs originates from the shape‐derived anisotropic polarizability of the rod‐like capsules.

Guided by the theoretical prediction of field‐parallel orientation, we next experimentally examined the alignment of UC RCs in a hydrogel precursor under an AC electric field (Figure 5). To preserve the field‐induced alignment after removing the electric field, UC RCs were dispersed in a hydrogel precursor solution consisting of acrylamide (monomer), N,N′‐methylenebisacrylamide (crosslinker), ammonium persulfate (initiator), and N,N,N′,N′‐tetramethylethylenediamine (accelerator). Upon applying an alternating current (AC) electric field across a 0.5 mm electrode gap (150 V_pp_, 300 kHz; amplified 50‐fold from 3 V_pp_), the UC RCs aligned into multiple chain‐like structures via a combination of dipole‐dipole interactions and dielectrophoretic (DEP) forces (Figure 5A). While dipolar interactions facilitated head‐to‐tail chaining, DEP forces guided the aligned chains toward each other in response to locally intensified field regions surrounding them [36, 37]. Concurrently, radical polymerization initiated by ammonium persulfate and N,N,N′,N′‐tetramethylethylenediamine fixed the aligned configuration of the UC RCs in the hydrogel matrix. Consequently, the field‐induced alignment of UC RCs was preserved within the polymerized hydrogel matrix (Figures 5B and Movie S1).

**FIGURE 5:**
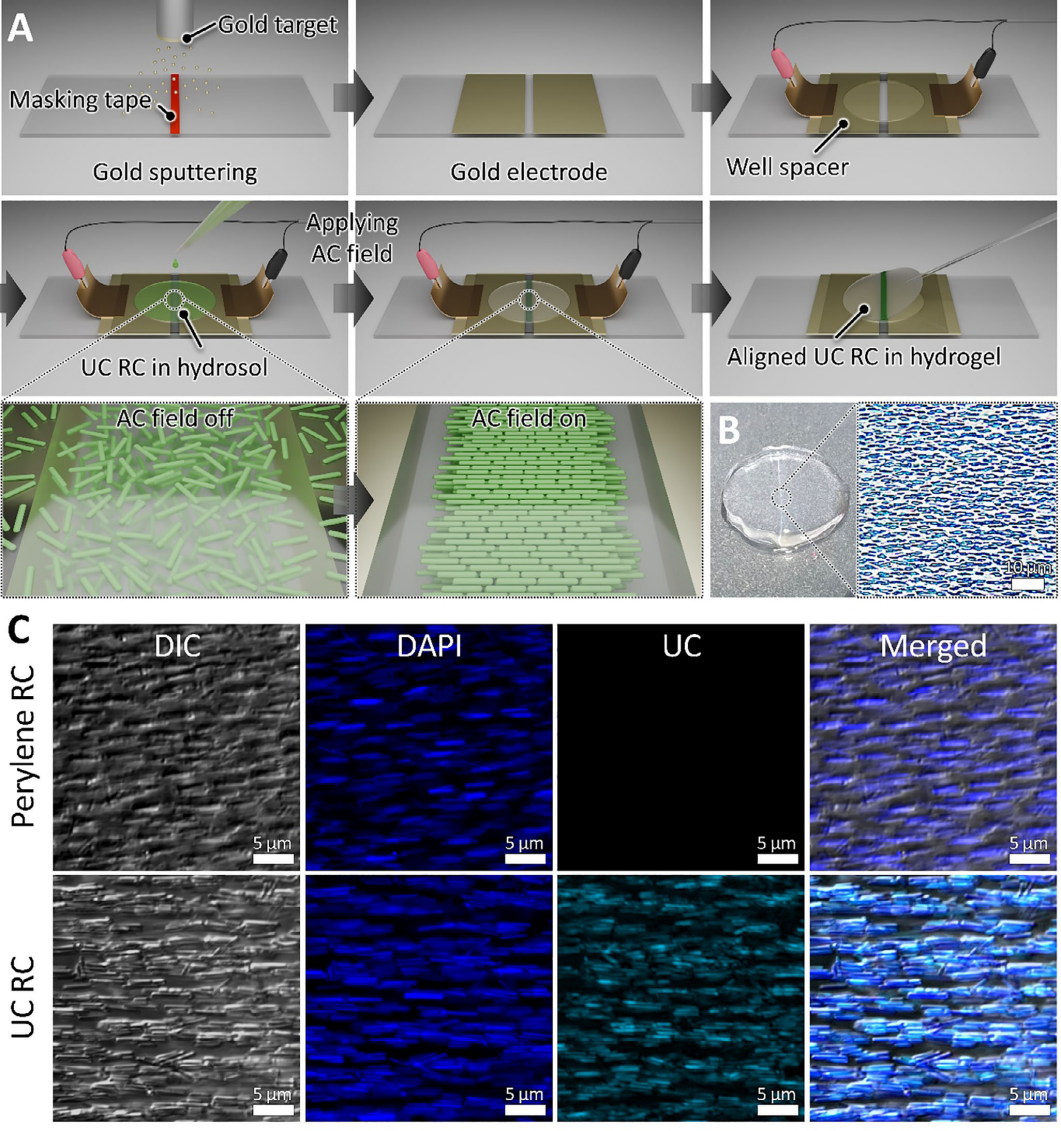
(A) Schematic illustration of the fabrication process of the aligned UC RCs in a hydrogel under an electric field. (B) Photograph of the hydrogel fabricated under an AC electric field (150 V_pp_, 300 kHz; amplified 50‐fold from 3 V_pp_) and corresponding bright‐field microscopy image showing the aligned UC RCs near the center. (C) CLSM images of aligned perylene RCs in hydrogel (top) and aligned UC RCs in hydrogel (bottom) in DIC, DAPI (λ_ex_ = 405 nm, λ_em_ = 400–600 nm), and UC (λ_ex_ = 640 nm, λ_em_ = 400–470 nm) channels.

The structural organization and optical properties of the UC RCs in the hydrogel were further examined using confocal laser scanning microscopy (CLSM) (Figure 5C). For comparison, perylene RCs without the sensitizer were prepared under identical conditions. Perylene RCs were visible under differential interference contrast (DIC) and DAPI (λ_*ex*_ = 405 nm, λ_*em*_ = 400–600 nm) channels but exhibited no signal in the UC channel (λ_*ex*_ = 640 nm, λ_*em*_ = 400–470 nm). This is because perylene RCs lack PdTPBP, which absorbs the 640 nm excitation light and sensitizes perylene triplet states; therefore, the TTA‐UC process cannot occur. In contrast, UC RCs exhibited strong blue upconverted emission in the UC channel and bright fluorescence in the DAPI channel. The fluorescence observed in the DAPI channel indicates the presence of perylene, whereas the UC‐channel emission confirms that the PdTPBP/perylene pair remained functionally active after hydrogel embedding and electric‐field alignment. Importantly, the UC‐channel signal followed the chain‐like arrangement observed in the DIC image, demonstrating that the field‐aligned UC RCs retained their upconversion capability within the hydrogel matrix.

To the best of our knowledge, this is the first demonstration of field‐aligned anisotropic TTA‐UC silica capsules embedded in a hydrogel matrix. Importantly, the fixed orientation of UC RCs may provide an additional optical parameter beyond TTA‐UC emission. Particle orientation can induce birefringence and polarization‐dependent optical responses, which have been utilized for information encoding and anti‐counterfeiting [38, 39]. Therefore, aligned UC RC hydrogels may provide a basis for optical authentication and anti‐counterfeiting materials that combine TTA‐UC emission with orientation‐dependent optical information.

## 3 Conclusion

We developed rod‐like TTA‐UC silica capsules by integrating hollow mesoporous silica rods with a sealed oil‐encapsulation strategy. HMSRs were prepared by coating silica rod templates with a mesoporous organosilica shell, followed by selective etching of the inner silica template. This all‐silica templating route enabled straightforward aspect‐ratio control while avoiding the use of metal or metal‐oxide rod templates. PdTPBP/perylene/squalene‐based TTA‐UC oil was subsequently infused into the hollow interior of HMSRs, and the open mesopores were sealed with an additional silica layer to obtain UC RCs. This capping process converted the open mesoporous carrier into a physically stable rod‐like capsule and effectively suppressed oil leakage during ethanol exposure and dry‐powder handling. The resulting UC RCs exhibited clear red‐to‐blue TTA‐UC in aqueous suspension. Power‐dependent measurements showed the characteristic quadratic‐to‐linear transition of TTA‐UC, with an experimental threshold intensity of 55 mW cm^−^^2^, and the upconversion quantum yield reached 1.47%. The functional significance of the rod‐like geometry was further examined through the field‐induced directional organization of the UC RCs. Coupled‐dipole calculations and rotational Monte Carlo simulations indicated that the shape‐derived anisotropic polarizability favors field‐parallel orientation, with an equilibrium mean angle of approximately 2.3° under the modeled field. Consistent with this predicted tendency, UC RCs were directionally organized in a hydrogel precursor under an AC electric field, and the resulting arrangement was preserved after polymerization. Confocal microscopy confirmed that the UC‐channel emission followed the chain‐like arrangement of UC RCs, demonstrating that their upconversion functionality was retained after hydrogel embedding and electric‐field alignment. Overall, UC RCs integrate stable oil encapsulation, efficient TTA‐UC emission, and anisotropy‐derived directional organization, suggesting their potential as versatile microcarriers for patterned luminescent materials, anti‐counterfeiting platforms, and other photonic applications requiring low‐intensity excitation and directional control.

## Author Contributions

**Jeong‐Min Park**: investigation, Writing – original draft, Writing – review and editing, methodology, validation, visualization. **Chanwook Cha**: writing – original draft, investigation, methodology, validation, writing – review and editing. **Hyun‐Seok Choe**: investigation, validation, visualization. **Geun Young Kim**: visualization, validation. **Suyoung Jeong**: visualization, validation. **Han‐Gil Kim**: methodology, validation, visualization. **Koohee Han**: validation, writing – review and editing, resources, methodology, funding acquisition. **Jae‐Hyuk Kim**: conceptualization, funding acquisition, writing – review and editing, supervision, resources, validation.

## Conflicts of Interest

The authors declare no conflicts of interest.

## Supporting information




**Supporting File 1**: advs78708‐sup‐0001‐SuppMat.docx.

**Supporting File 2**: advs78708‐sup‐0002‐MovieS1.mp4.

## Data Availability

The data that support the findings of this study are available from the corresponding author upon reasonable request.
