# Supplementary material for: Triplet‐Triplet Annihilation‐Based Upconversion in Rod‐Like Silica Capsules
Source: Adv Sci (Weinh). 2026 Nov 9:e78708. Online ahead of print. doi: 10.1002/advs.78708 (PMC13748911; doi:10.1002/advs.78708)
Supplement: Supplementary file 1 [file ADVS-9999-e78708-s002.docx]

Supporting Information

Title: Triplet-Triplet Annihilation-Based Upconversion in Rod-Like Silica Capsules

*Jeong-Min Park^1†^, Chanwook Cha^2†^, Hyun-Seok Choe^3^, Geun Young Kim^1, 4^, Suyoung Jeong^1^, Han-Gil Kim^1^, Koohee Han^2*^, and Jae-Hyuk Kim^1, 4*^*

^1^Department of Civil and Environmental Engineering, Pusan National University, Busan, 46241, Republic of Korea

E-mail: jaehyuk.kim@pusan.ac.kr

^2^School of Chemical Engineering and Applied Chemistry, Kyungpook National University, Daegu, 41566, Republic of Korea

E-mail: han.koohee@knu.ac.kr

^3^Center for Advanced Specialty Chemicals, Korea Research Institute of Chemical Technology, Ulsan, 44412, Republic of Korea

^4^Institute for Environment and Energy, Pusan National University, Busan, 46241, Republic of Korea

† Jeong-Min Park and Chanwook Cha contributed equally to this work.

1. Materials

Acetic acid (99.7%), ammonia solution (28%), ammonium nitrate (NH_4_NO_3_), ethanol, methanol, pentanol, and sodium hydroxide (NaOH) were purchased from Samchun Chemicals (Republic of Korea). Acrylamide, ammonium persulfate (≥98.0%), 1,2-bis(triethoxysilyl)ethane (BTSE), cetyltrimethylammonium bromide (CTAB), N,N'-methylenebisacrylamide, N,N,N',N'-tetramethylethylenediamine (99%), polyvinylpyrrolidone (PVP, average molecular weight: 55,000), tetraethyl orthosilicate (TEOS), and trisodium citrate dihydrate (TSC) were purchased from Sigma-Aldrich. Perylene and squalene were purchased from Tokyo Chemical Industry. Palladium(II) meso-tetraphenyltetrabenzoporphyrin (PdTPBP) was synthesized as described previously.^[S1]^ All experiments used ultrapure water with a resistivity of 18.2 MΩ·cm. All chemicals were used as received without further purification.

2. Preparation of SR

SR was synthesized following a modified procedure described in the literature.^[S2]^ A PVP/pentanol solution was prepared by mixing PVP (10 g) with pentanol (100 mL). In a 50 mL conical tube, 27.5 mL of the PVP/pentanol solution was mixed with 2.5 mL of ethanol. Subsequently, an aqueous TSC solution (6.1 mM, 480 μL), TEOS (300 μL), and deionized water (550 μL) were added to the mixture. The mixture was then vigorously shaken by hand. Subsequently, 550 μL of ammonia solution was added, and the mixture was gently mixed and incubated in a 35 °C water bath for 18 h. The resulting mixture was washed by centrifugation (3075 rcf, 4 min) several times with water, 10% acetic acid in ethanol, and ethanol, sequentially. To further purify the particles, the mixture was subjected to additional centrifugation steps at a lower speed (400 rcf, 7 min). Finally, the supernatant was removed, and the resulting powder was dried in an oven at 60 °C.

3. Preparation of HMSR

To coat the SR with a mesoporous organosilica layer, a batch of dried SR was dispersed in a methanol-water solution (40 mL, 38.75:61.25). Then, 400 μL of ammonia solution and 1 mL of CTAB solution (1 g of CTAB in 10 mL of methanol) were added to the mixture. The mixture was then rotated at 3 rpm. After 10 min, 60 μL of BTSE was added, and the rotation was continued at 3 rpm. After 2 h, 500 μL of CTAB solution was added, and the mixture was rotated for 10 min. Then, 60 μL of BTSE was added, and the mixture was rotated for an additional 2 h to obtain SR@mSiO_2_. To remove the CTAB template, the mixture was centrifuged (3075 rcf, 4 min), and the precipitate was resuspended in 10% acetic acid (in ethanol) and sonicated at 60 °C for 30 min. After centrifugation, the precipitate was resuspended in an ethanolic NH_4_NO_3_ solution (6 g of NH_4_NO_3_ in 950 mL of ethanol and 50 mL of water) and sonicated at 60 °C for 30 min. The particles were then washed several times with ethanol and water by centrifugation. Finally, the CTAB-extracted SR@mSiO_2_ was dispersed in water (40 mL). To etch the inner SR, the SR@mSiO_2_ suspension was heated to 70 °C, and 2 mL of 0.5 M NaOH solution was added. The reaction was allowed to proceed for 20 min. The HMSR suspension was then collected by filtration using a 1.2 μm PES membrane, washed several times with ethanol and water, and dried in a convection oven at 60 °C.

4. Preparation of UC HMSR and UC RC

The UC oil (PdTPBP/perylene/squalene) was prepared by dissolving PdTPBP (1.68 mg) and perylene (20 mg) in squalene (10 mL), followed by filtration through a PTFE syringe filter to remove any precipitate. For UC oil encapsulation, a batch of dried HMSRs were mixed with UC oil (1 mL), sonicated for 10 min, and stirred vigorously at 1000 rpm on a 60 °C hot plate for 1 h. Heating was stopped after confirming that the UC oil had infused into the HMSRs (UC HMSRs) using a microscope. Next, water (4 mL) and ammonia solution (100 μL) were added, and the mixture was vigorously stirred at 1000 rpm for 30 min to migrate the UC HMSRs from the UC oil to the water phase. Finally, the aqueous layer was carefully extracted using a pipette, centrifuged at 5000 rcf for 4 min, and washed twice with water to remove any remaining UC oil emulsions.

The resulting UC HMSRs were then dispersed in water (10 mL). To obtain UC RCs via the pore-capping process, the suspension was transferred to a 20 mL vial. Ammonia solution (200 μL) and TEOS (250 μL) were added, and the mixture was stirred overnight on a hot plate at 50 °C. The resulting UC RCs were collected by centrifugation, washed several times with ethanol and water, and dried for storage. Control capsules, including squalene-laden RCs, PdTPBP RCs, and perylene RCs, were prepared using the same encapsulation and pore-capping procedure, except that the UC oil was replaced with squalene, PdTPBP/squalene, or perylene/squalene, respectively.

5. Alignment of UC RCs in a hydrogel

For electrode fabrication, a 0.5 mm tape was applied to a glass substrate to define the electrode pattern. The substrate was then placed inside an e-beam evaporator (KVE-2000, Korea Vacuum Tech Co.) equipped with a 7.1 kW electron gun for metal deposition, and the tape was removed. The AC field was generated using a function generator (DZ1022Z), amplified 50-fold using a power amplifier (WMA-300), and the applied voltage was monitored using a digital multimeter (DM3058E). The hydrogel precursor solution was prepared by dissolving 1.1 g of acrylamide and 0.010 g of N,N'-methylenebisacrylamide in 20 mL of DI water. For the alignment experiment, several drops of the UC RC suspension were dispersed in the precursor solution. Polymerization was initiated by adding 20 µL of a 10 wt% ammonium persulfate solution and 2 µL of N,N,N',N'-tetramethylethylenediamine. The final mixture was immediately placed on the patterned electrodes. The alignment behavior of the UC RCs under the applied AC field was observed in real time using an optical microscope (BX52, Olympus) equipped with a charge-coupled device (CCD) camera (DP28, Olympus) as the hydrogel was polymerized.

6. Simulation of field-induced rod alignment

Rotational Monte Carlo simulations were performed to evaluate the field-induced orientational relaxation of silica rods. The simulations were based on the coupled-dipole method (CDM) reported by Troppenz et al., using the experimentally measured rod geometry and an electric-field magnitude calculated from the applied AC voltage. Randomly oriented rods were subjected to rotational trial moves according to a Metropolis Monte Carlo scheme, and their relaxation toward the electric-field direction was analyzed. Detailed equations, model assumptions, parameters, and calculation procedures are provided in Text S3.

7. Characterization

The morphologies of the synthesized materials were investigated using a field-emission transmission electron microscope (FE-TEM; TALOS F200X, Thermo Fisher Scientific Co., USA) and a field-emission scanning electron microscope (FE-SEM; Supra 25, Zeiss, Germany). High-angle annular dark-field STEM (HAADF-STEM) imaging and energy-dispersive X-ray spectroscopy (EDS) elemental mapping were performed using a TALOS F200X to confirm the encapsulated components. An X’Pert3 diffractometer (Malvern Panalytical Ltd., UK) was used to measure the X-ray diffraction (XRD) pattern using Cu Kα (40 kV and 40 mA) radiation. A Tristar II Plus (Micromeritics Instrument Co., USA) was used to measure the nitrogen adsorption/desorption isotherms and pore volume. A spectrofluorometer (FS5-TCSPC, Edinburgh Instruments, Edinburgh, UK) fitted with a 635 nm commercial diode laser was used to obtain the absorbance and emission spectra, phosphorescence, and TTA-UC properties of the samples. The incident laser intensity was measured using a power meter (834-R, Newport Corporation, Irvine, CA, USA) to calculate the power density, and a 632 nm notch filter was used to remove the scattered laser light. A fluorescence microscope (Eclipse Ni-U, Nikon, Japan) was used to acquire bright-field and fluorescence images. Specific TTA-UC imaging was acquired using a customized setup based on a fluorescence microscope, utilizing a 635 nm laser and appropriate optical filters. A confocal laser scanning microscope (CLSM) (LSM 800, Carl Zeiss Co., Germany) was used to acquire the CLSM images.

Figure S1. Schematic illustration of the synthetic process for HMSRs.

Figure S2. XRD patterns of HMSRs.

Figure S3. (A) Nitrogen sorption isotherm and (B) pore size distribution curve of HMSRs.

Figure S4. SEM images of HMSRs with different aspect ratios obtained by varying the amount of TEOS.

Figure S5. Schematic energy diagram illustrating the TTA-UC photochemical process, including absorption (635 nm), intersystem crossing (ISC), triplet-triplet energy transfer (TTET), triplet-triplet annihilation (TTA), and delayed upconverted emission (470 nm).

Figure S6. Schematic illustration and corresponding photographs of the synthetic process of UC RCs from HMSRs.

Figure S7. Photographs comparing UC HMSRs (control) and UC RCs (capped) immediately after filtration (left) and after drying (right).

Figure S8. Normalized absorption (ABS) (solid lines) and photoluminescence (PL) (dashed lines) for perylene (blue) and PdTPBP (red) in squalene.

Figure S9. Excitation-power-dependent UC emission decay profiles of UC RCs. The decay profiles were globally fitted using $\boldsymbol{I}_{\mathbf{UC}}\left( \boldsymbol{t} \right)\boldsymbol{=}\boldsymbol{I}_{\mathbf{UC}}\left( \boldsymbol{0} \right)\left[ \left( \boldsymbol{1-\beta} \right)\boldsymbol{/}\left( \exp\left( \boldsymbol{t/}\boldsymbol{\tau}_{\boldsymbol{T}} \right)\boldsymbol{-\beta} \right) \right]^{\boldsymbol{2}}$, where $\boldsymbol{\tau}_{\boldsymbol{T}}$ is the perylene triplet lifetime and $\boldsymbol{\beta}$ represents the relative contribution of bimolecular TTA to the initial triplet depopulation.^[S3]^ A common $\boldsymbol{\tau}_{\boldsymbol{T}}$ was used for all excitation intensities, while $\boldsymbol{\beta}$ was independently fitted for each decay profile.

Text S1. UCQY measurement

The UCQY (Φ_UC_) of the UC RCs ([PdTPBP] = 0.183 mM and [perylene] = 7.93 mM) was measured relative to the phosphorescence quantum yield of PdTPBP RCs ([PdTPBP] = 0.183 mM) in response to laser excitation at 635 nm (Φ_std_=7%, measured using C9920−02G instrument, Hamamatsu) and calculated using the equation below.

$$\begin{aligned} \Phi_{UC}=\Phi_{std}\left( \frac{I_{UC}}{I_{std}} \right)\left( \frac{1-{10}^{-A_{std}}}{1-{10}^{-A_{UC}}} \right)\left( \frac{\eta_{UC}}{\eta_{std}} \right)^{2} \end{aligned}$$

I, A, and η denote the integrated photoluminescence profile, absorbance at 635 nm, and refractive index of the media, respectively. The subscripts “std” and “UC” refer to the phosphorescence of the reference standard (PdTPBP RCs, integrated over 750–850 nm) and the UC emission of the UC RCs (integrated over 420–600 nm), respectively. In this calculation, the absorbance and refractive index of the medium are identical because both samples were synthesized using an identical synthetic procedure.

Text S2. Calculation of the theoretical surface-to-volume (S/V) ratio

To quantitatively compare the geometrical characteristics of the UC RC with conventional TTA-UC nanoparticles, the theoretical surface-to-volume (S/V) ratio of the UC RC was calculated based on its dimensions. The geometrical model of the UC RC was defined as a cylinder with two hemispherical ends. From the morphological analysis by SEM images, the outer diameter and total length of the UC RC were set to 426 nm (radius, $R$ = 213 nm) and 3.64 µm (total length, $L_{total}$ = 3640 nm), respectively. The length of the purely cylindrical part ($L_{cyl}$) is calculated as:

$$L_{cyl}=L_{total}-2R=3214\text{ nm}$$

The total outer surface area ($A_{rod}$) and volume ($V_{rod}$) of the UC RC were calculated using the following equations:

$$A_{rod}=2\pi RL_{cyl}+4\pi R^{2}\approx4,870,886 nm^{2}$$

$$V_{rod}=\pi R^{2}L_{cyl}+\frac{4}{3}\pi R^{3}\approx498,477,624 nm^{3}$$

Consequently, S/V ratio of UC RC = $A_{rod}/V_{rod}$ ≈ 0.00977 nm⁻¹.

For comparison, the surface area ($A_{sph}$) and volume ($V_{sph}$) of a theoretical spherical nanoparticle with a diameter of 200 nm (radius, $r$ = 100 nm) were calculated as follows:

$$A_{sph}=4\pi r^{2}\approx125,664\text{ nm}^{2}$$

$$V_{sph}=\frac{4}{3}\pi r^{3}\approx4,188,790\text{ nm}^{3}$$

S/V ratio of 200 nm sphere = $A_{sph}/V_{sph}$ = 0.03 nm⁻¹.

This geometrical calculation demonstrates that the theoretical S/V ratio of the micrometer-scale UC RC (0.00977 nm⁻¹) is approximately 32.5% of that of a conventional 200 nm isotropic spherical nanoparticle (0.03 nm⁻¹).

Text S3. Coupled dipole method (CDM) and rotational Monte Carlo simulation

In the CDM calculation, the effective rod was represented by $N$ polarizable units located at fixed lattice sites on a cubic grid ($N=401$). The experimentally measured rod dimensions and the dielectric properties of the effective rod and surrounding medium were used in the calculation. The dipole moment of each polarizable unit, $\mathbf{p}_{i}$, was assumed to depend linearly on the local electric field acting on that unit, $\mathbf{E}_{\mathrm{loc}}^{\left( i \right)}$:

$$\mathbf{p}_{i}=\alpha\mathbf{E}_{\mathrm{loc}}^{\left( i \right)}$$

Here, $\alpha$ is the excess polarizability of each polarizable unit, which is given by the Clausius-Mossotti relation:

$$\alpha=\frac{3}{4\pi}\frac{\varepsilon_{2}}{n_{0}}\frac{\varepsilon_{1}-\varepsilon_{2}}{\varepsilon_{1}+2\varepsilon_{2}}$$

In this expression, $\varepsilon_{1}$ and $\varepsilon_{2}$ are the dielectric constants of the effective rod and the surrounding medium, respectively, and $n_{0}$ denotes the number density of dipoles in the rod. The dipole-dipole coupling between polarizable units is described using an interaction tensor ($\mathbf{T}_{ij}$), which depends on the relative position vector between unit $i$ and unit $j$ ($\mathbf{r}_{i}-\mathbf{r}_{j}$).

$$\mathbf{T}\left( \mathbf{r} \right)=\left\{ \begin{matrix} \frac{3\hat{\mathbf{r}}\hat{\mathbf{r}}-\mathbf{I}}{\varepsilon_{2}\left| \mathbf{r} \right|^{3}}, & \mathbf{r}\neq0 \\ 0, & \mathbf{r}=0 \end{matrix} \right.$$

Here, $\hat{\mathbf{r}}=\mathbf{r}/\mid\mathbf{r}\mid$ is the unit vector along $\mathbf{r}$, $\mathbf{I}$ is the $3\times3$ identity matrix, and the null tensor for $\mathbf{r}=0$ excludes self-interaction. A dipole at position $\mathbf{r}_{j}$ generates an induced electric field at position $\mathbf{r}_{i}$, and the local electric field acting on unit $i$ ($\mathbf{E}_{\mathrm{loc}}^{\left( i \right)}$) can therefore be written as:

$$\mathbf{E}_{\mathrm{loc}}^{\left( i \right)}=\mathbf{E}+\sum_{j=1}^{N} \mathbf{T}_{ij}\mathbf{p}_{j}$$

In this equation, $\mathbf{E}$ is the external electric field, $\mathbf{p}_{j}$ is the dipole moment of unit $j$, and $\mathbf{T}_{ij}$ is shorthand for $\mathbf{T}(\mathbf{r}_{i}-\mathbf{r}_{j})$. Combining the linear dipole response with the local-field expression gives the coupled dipole equation for unit $i$:

$$\mathbf{p}_{i}=\alpha\mathbf{E}+\alpha\sum_{j=1}^{N} \mathbf{T}_{ij}\mathbf{p}_{j}$$

Consequently, the coupled dipole equations can be rewritten in a compact matrix form as:

$$\left( \mathcal{I}-\alpha\mathcal{T} \right)\mathcal{P}=\alpha\mathcal{E}$$

Here, $\mathcal{P}$ is a $3N$-dimensional vector containing all dipole moment vectors $\mathbf{p}_{i}$, $\mathcal{E}$ contains $N$ copies of the external electric field $\mathbf{E}$, $\mathcal{T}$ is the $3N\times3N$ block interaction matrix composed of $\mathbf{T}_{ij}$, and $\mathcal{I}$ is the corresponding identity matrix. Solving this linear system gives the induced dipole moments of all polarizable units in the rod. The collective excess polarizability tensor of the rod, $\boldsymbol{\alpha}_{c}$, is then obtained from the relation:

$$\sum_{i=1}^{N} \mathbf{p}_{i}=\boldsymbol{\alpha}_{c}\cdot\mathbf{E}$$

Diagonalization of $\boldsymbol{\alpha}_{c}$ gives two principal polarizabilities, $\alpha_{c,\parallel}$ and $\alpha_{c,\perp}$, corresponding to the directions parallel and perpendicular to the long axis of the rod, respectively. Because these two principal polarizabilities are different for an anisotropic rod-like particle, the field-induced potential energy depends on the orientation angle $\theta$, defined relative to the electric-field direction.

The electric field experimentally applied across the hydrogel precursor was sinusoidal. Its root-mean-square magnitude was calculated from the amplified peak-to-peak voltage, $V_{\mathrm{pp}}$, and electrode gap, $d$:

$$E=\frac{V_{pp}}{2\sqrt{2}d_{gap}}$$

Rotational Monte Carlo simulations were then performed using the CDM-derived orientation-dependent excess potential energy. The rods were initially assigned random three-dimensional orientations. Trial rotational moves were repeatedly proposed and accepted or rejected according to the Metropolis criterion described in the main text. One Monte Carlo sweep was defined as one attempted rotational move per rod. This rotational Monte Carlo simulation was designed to isolate the field-induced orientational bias of individual noninteracting rods. Interparticle interactions, translational motion, hydrodynamic and wall effects, dielectrophoretic translation, chain formation, and hydrogel polymerization dynamics were not included.

Table S1. Key parameters used for the field-induced orientational relaxation calculation of silica rods.

| Parameter | Meaning | Value |
| --- | --- | --- |
| $L$ | Rod length | $3.64 \mu m$ |
| $D$ | Rod diameter | $0.426 \mu m$ |
| $L/D$ | Rod aspect ratio | $8.54$ |
| $\varepsilon_{1}$ | Relative permittivity of silica | $3.9$ |
| $\varepsilon_{2}$ | Relative permittivity of medium | $78$ |
| $n_{0}$ | Number density of the dipoles in the rod | $8.04\times{10}^{20} m^{-3}$ |
| $V_{pp}$ | Amplified peak-to-peak voltage | $150 V$ |
| $d_{gap}$ | Electrode gap | $0.5 mm$ |
| $E$ | Root-mean-square electric field | $0.106 V{\mu m}^{-1}$ |
| $T$ | Temperature | $298.15 K$ |
| $k_{B}$ | Boltzmann constant | $1.38\times{10}^{-23} JK^{-1}$ |
| $\alpha_{c, \parallel}$ | Cluster polarizability along longitudinal axis | $-3.28\times{10}^{-28}Cm^{2}V^{-1}$ |
| $\alpha_{c, \perp}$ | Cluster polarizability along transverse axis | $-6.81\times{10}^{-28}Cm^{2}V^{-1}$ |

**References**
[S1] X. Cui, J. Zhao, P. Yang, J. Sun, *Chem. Commun.* **2013**, *49*, 10221.

[S2] A. Kuijk, A. Van Blaaderen, A. Imhof, *Journal of the American Chemical Society* **2011**, 133, 2346.

[S3] F. Edhborg, A. Olesund, B. Albinsson, *Photochemical & Photobiological Sciences* **2022**, 21, 1143.
